# Common Proprotein Convertase Subtilisin/Kexin Type 9 (PCSK9) Epitopes Mediate Multiple Routes for Internalization and Function

**DOI:** 10.1371/journal.pone.0125127

**Published:** 2015-04-23

**Authors:** Rachel M. DeVay, Lynn Yamamoto, David L. Shelton, Hong Liang

**Affiliations:** Rinat-Pfizer Inc., South San Francisco, California, United States of America; Centro Cardiologico Monzino, ITALY

## Abstract

Proprotein convertase subtilisin/kexin type 9 (PCSK9) is a soluble protein that directs membrane-bound receptors to lysosomes for degradation. In the most studied example of this, PCSK9 binding leads to the degradation of low density lipoprotein receptor (LDLR), significantly affecting circulating LDL-C levels. The mechanism mediating this degradation, however, is not completely understood. We show here that LDLR facilitates PCSK9 interactions with amyloid precursor like protein 2 (APLP2) at neutral pH leading to PCSK9 internalization, although direct binding between PCSK9 and LDLR is not required. Moreover, binding to APLP2 or LDLR is independently sufficient for PCSK9 endocytosis in hepatocytes, while LDL can compete with APLP2 for PCSK9 binding to indirectly mediate PCSK9 endocytosis. Finally, we show that APLP2 and LDLR are also required for the degradation of another PCSK9 target, APOER2, necessitating a general role for LDLR and APLP2 in PCSK9 function. Together, these findings provide evidence that PCSK9 has at least two endocytic epitopes that are utilized by a variety of internalization mechanisms and clarifies how PCSK9 may direct proteins to lysosomes.

## Introduction

High serum LDL-cholesterol (LDL-C) levels correlate strongly with hypercholesterolemia and coronary artery disease (CAD). Thus, multitudes of CAD prevention therapeutics focus on lowering LDL-C levels. One such approach aims to increase expression of the LDL receptor (LDLR), a protein that clears LDL-C from the blood. LDL binds LDLR on the cell surface, and following internalization, LDLR undergoes a pH-dependent conformational change upon entering endosomes. This causes LDLR to release bound LDL which is then delivered to lysosomes, while LDLR itself is recycled back to the cell surface to repeat the process [1].

PCSK9 is a soluble, secreted protein that regulates LDLR protein levels by binding LDLR on the plasma membrane and directing it towards lysosomes [2–5]. In addition to LDLR, PCSK9 mediates lysosomal degradation of a number of receptors, including very low density lipoprotein receptor (VLDLR), Apolipoprotein E receptor 2 (APOER2), and Beta secretase 1 (BACE1) [6–9]. PCSK9 likely utilizes its c-terminal Cis-His Rich Domain (CHRD) to mediate post-endocytic lysosomal delivery of its targets [2, 10–13]. Importantly, the CHRD interacts in a pH dependent manner with APLP2, a member of the amyloid precursor protein (APP) family. This interaction allows PCSK9 to bridge LDLR to APLP2, which in turn transports the entire complex to lysosomes [14].

Human genetics studies demonstrate that people who harbor loss of function PCSK9 mutations have low LDL-C levels and decreased risk of CAD [15, 16], while gain of function PCSK9 carriers show the opposite effects [17, 18]. PCSK9 has therefore become a promising target for treating hypercholesterolemia. Indeed, LDL-C can be effectively attenuated using monoclonal antibody therapeutics against PCSK9 that inhibit its interactions with LDLR [19–24]. We previously reported one such PCSK9 neutralizing antibody, J16, which lowers serum LDL-C in rodents and non-human primates [19, 22].

J16 has a short, dose dependent half-life at low doses, indicating that it undergoes target mediated clearance [19]. Indeed, we observed that J16, when bound to PCSK9, is directed to lysosomes in the same manner as LDLR. [14, 19]. These findings were somewhat surprising since PCSK9 endocytosis has been shown to be dependent on its binding to LDLR [25, 26], and J16 abolishes measurable LDLR/PCSK9 interactions. We found, however, that LDLR expression is necessary for PCSK9/J16 internalization [14]. Thus, in the absence of a direct interaction, LDLR may still play a critical regulatory role in PCSK9 endocytosis. In this study, we sought to elucidate the mechanism(s) of PCSK9 internalization that are independent of direct LDLR binding in hepatic cells.

## Results

### APLP2 and LDLR mediate PCSK9 internalization *in vitro*

Numerous reports in the literature have shown that LDLR mediates PCSK9 endocytosis [25, 26]. We determined in a previous study that PCSK9 has additional internalization mechanisms that are independent of LDLR binding [14]. While we showed novel interactions between PCSK9 and APP or APLP2, our data indicated that neither of these interactions was required for PCSK9 endocytosis. We therefore hypothesize here that LDLR, APP, or APLP2 may be independently sufficient to mediate PCSK9 internalization, which would allow them to compensate for each other.

To better understand PCSK9 endocytosis and dissect the individual or combined roles of LDLR, APLP2 or APP, we used two different monoclonal antibodies that bind PCSK9; J16, that specifically inhibits PCSK9 binding to LDLR, and 5F6, that specifically blocks PCSK9 binding to APLP2 and APP. We then subjected J16 and 5F6 in various combinations to our previously validated internalization assay [14] in an effort to identify compensatory endocytic mechanisms amongst these players.

**Fig 1:**
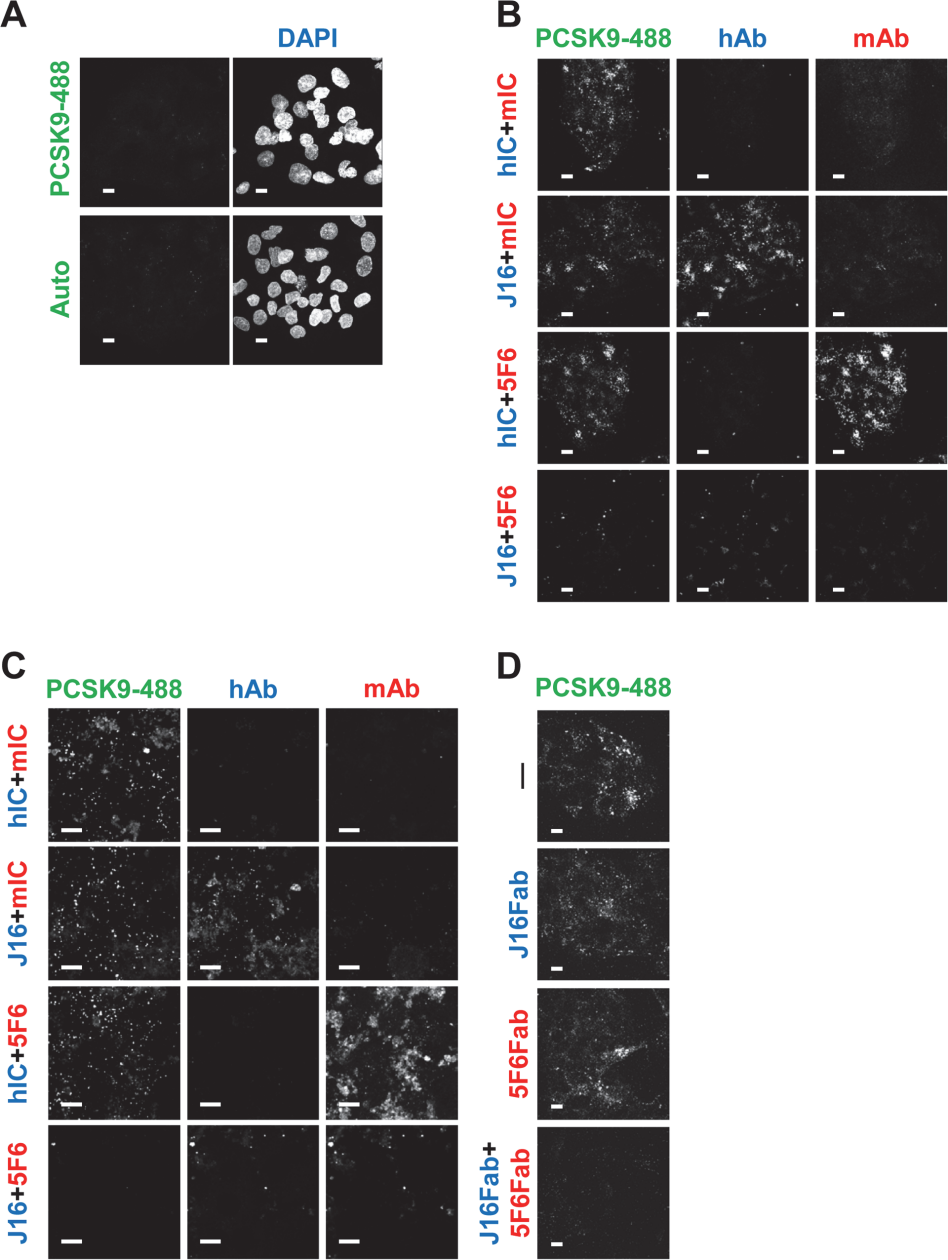
PCSK9 internalization with 5F6, J16, or combination of both. (A) PCSK9-488 cell surface binding performed identically to internalization assays, only at 4 degrees (top). Autofluorescence of HepG2 cells shown on bottom. (B and C) PCSK9-488 internalization in the presence of mouse isotype control (mIC) and human isotype control (hIC) antibodies (top row), mIC and J16 (middle top row), hIC and 5F6 (middle bottom row), or 5F6 and J16 (bottom row) in (B) HepG2 cells or (C) primary mouse hepatocytes. Internalized human and mouse antibodies shown in blue and red, respectively. (D) PCSK9-488 internalization alone (top), in the presence of J16 Fab (middle top), 5F6 Fab (middle bottom), or 5F6 Fab and J16 Fab (bottom). Scale bars, 10 μM. All experiments were performed independently at least 3 times and representative data are shown here.

Recombinant human PCSK9 conjugated with Alexa Fluor 488 (PCSK9-488) was premixed with the following combinations of antibodies: (1) mouse isotype control antibody (mIC) + human isotype control antibody (hIC), (2) J16 + mIC, (3) hIC + 5F6, or (4) J16 + 5F6. All antibodies were used at a molar excess to saturate PCSK9-488, and the complexes were monitored for internalization in HepG2 cells or mouse primary hepatocytes. Importantly, these conditions were previously optimized to ensure fluorescence signal as detected by a confocal microscope from surface bound PCSK9-488 is negligible (Fig 1A) [14]. Thus, the majority of observed signal is attributable to internalized PCSK9. Consistent with our previous report that PCSK9 can be internalized in the absence of direct interactions with LDLR, APP, or APLP2, PCSK9-488 complexed with J16 or 5F6 alone was endocytosed in both HepG2 cells and primary mouse hepatocytes as efficiently as PCSK9-488 combined with mIC and hIC antibodies (Fig 1B and 1C) [14]. However, internalization of PCSK9-488 bound by both 5F6 and J16 was significantly inhibited (Fig 1B and 1C). Similarly, the combination of 5F6 and J16 Fab fragments blocked PCSK9 endocytosis in HepG2 cells, while either Fab alone had no appreciable effect (Fig 1D), negating the possibility that binding of two IgG molecules sterically hindered PCSK9 internalization.

**Fig 2:**
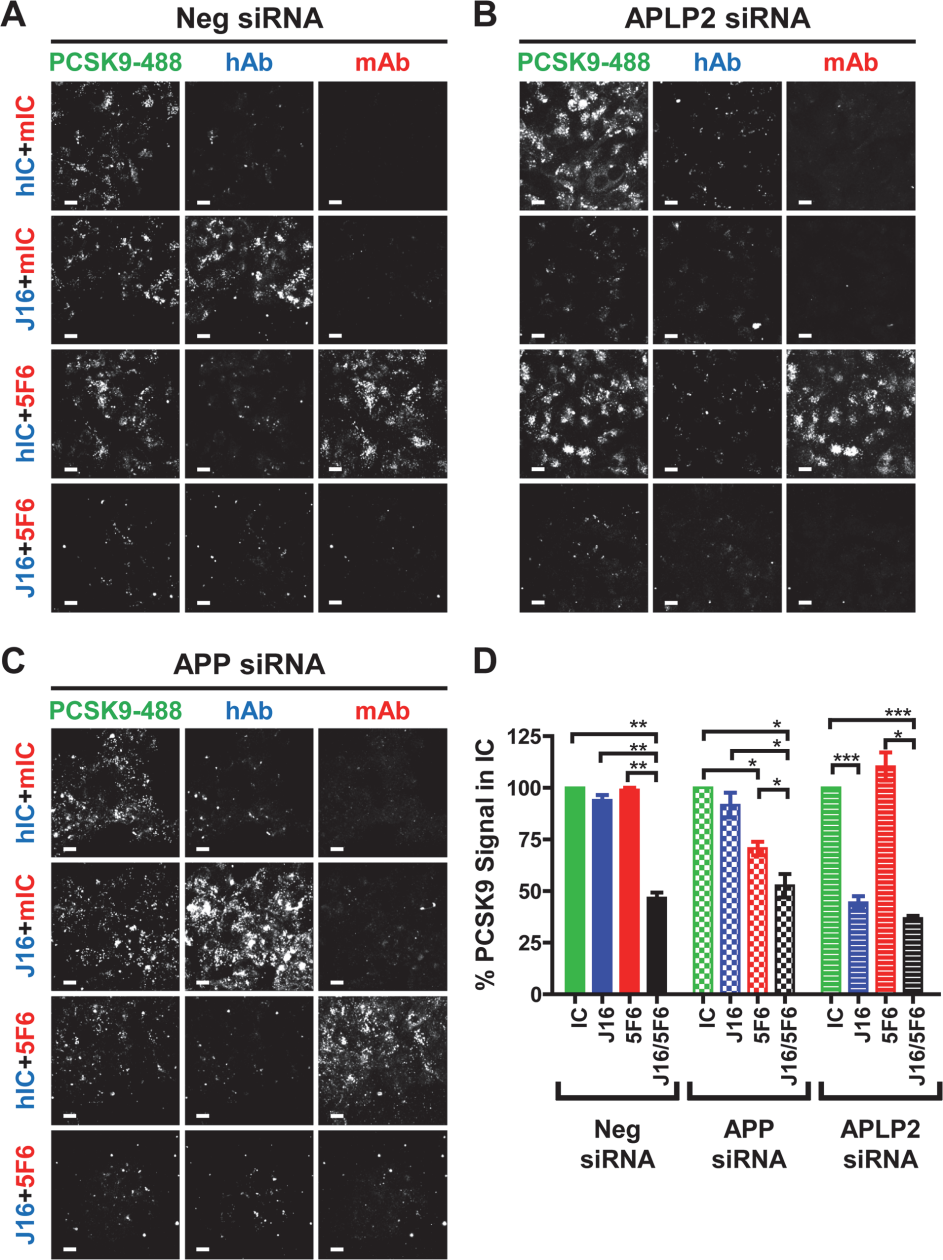
Determining which of APP or APLP2 affects PCSK9 internalization. (A, B, and C) PCSK9-488 internalization in the presence of mIC and hIC antibodies (top row), mIC and J16 (middle top row), hIC and 5F6 (middle bottom row), or 5F6 and J16 (bottom row) in (A) Neg, (B) APLP2, or (C) APP siRNA treated HepG2 cells. Internalized human and mouse antibodies shown in blue and red, respectively. Scale bars, 10 μM. (D) Quantification of (A, B, and C) shown as average fluorescent signal of PCSK9-488 per cell normalized to IC with SEM of 3 independent experiments.

**Fig 3:**
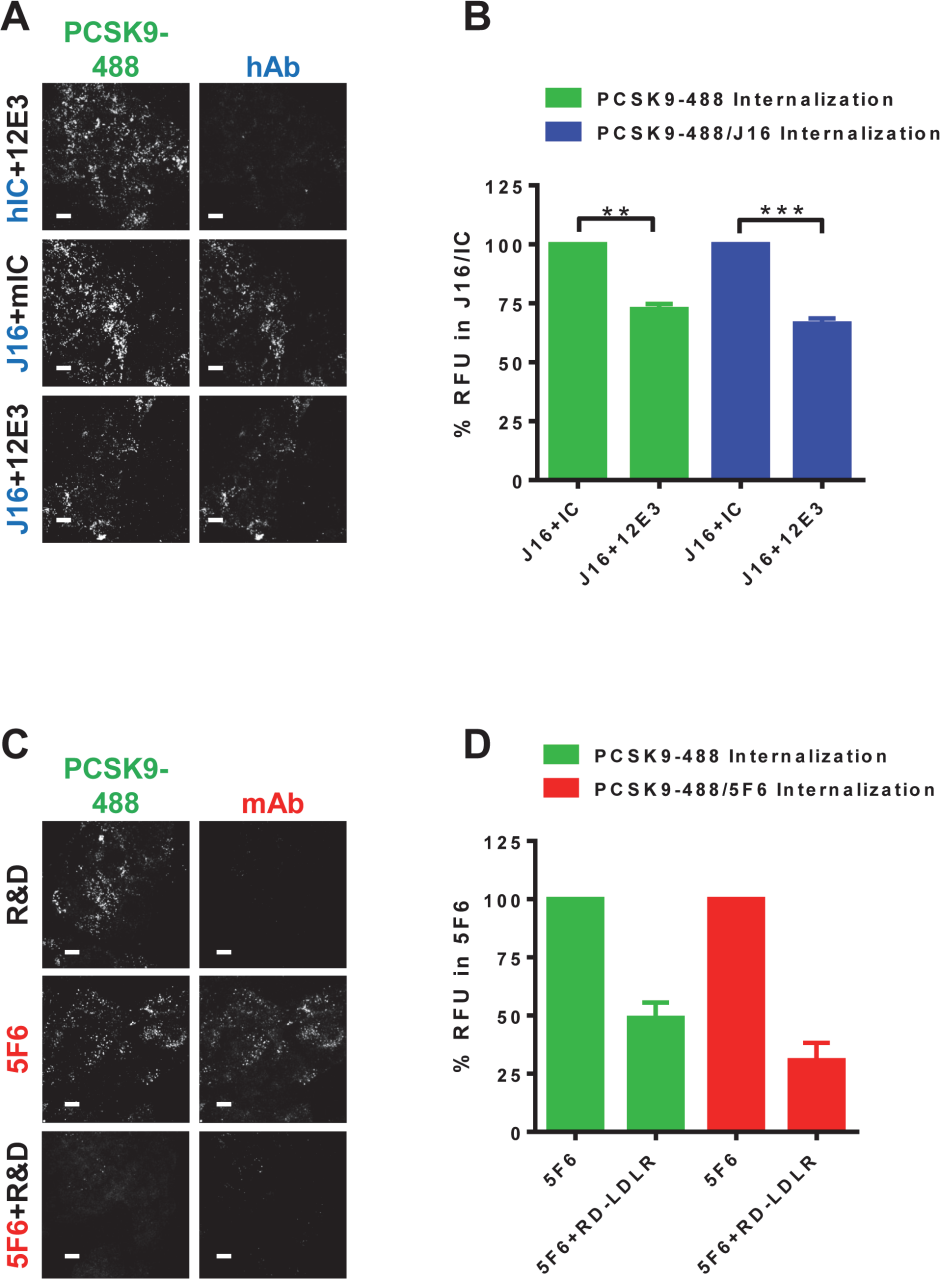
Anti-APLP2 or Anti-LDLR antibody effects on PCSK9 internalization. (A) PCSK9-488 internalization in the presence of 12E3 and hIC (top row), mIC and J16 (middle row) or 12E3 and J16 (bottom row). Internalized human antibodies shown in blue. Scale bars, 10 μM. (B) Quantification of (A) shown as average percent signal of PCSK9-488 per cell of PCSK9-488/J16 (green bars) or average percent signal of J16 per cell of PCSK9-488/J16 (blue bars) with SEM from 3 independent experiments. (C) PCSK9-488 internalization in the presence of RD-LDLR (top row), hIC and 5F6 (middle row) or RD-LDLR and 5F6 (bottom row). Internalized mouse antibodies shown red. Scale bars, 10 μM. (D) Quantification of (C) shown as average percent signal of PCSK9-488 per cell of PCSK9-488/5F6 (green bars) or average percent signal of 5F6 per cell of PCSK9-488/5F6 (red bars) with SEM from 3 independent experiments.

We next tested PCSK9 internalization in HepG2 cells whose protein expression of APLP2 or APP was lowered by 89% or 87% respectively using specific siRNA oligos (S1A Fig). These oligos were previously tested in internalization assays to rule out off-target effects [14]. As expected, loss of either APP or APLP2 did not alter PCSK9 endocytosis relative to Negative control (Neg) cells, and PCSK9-488 internalization was significantly blocked when complexed with both J16 and 5F6 under all conditions (Fig 2A, 2B, 2C and 2D). Interestingly, J16 significantly inhibited PCSK9-488 internalization in *APLP2*, but not *APP* or Neg, siRNA treated cells (Fig 2A, 2B, 2C and 2D), indicating that APLP2 but not APP is required for PCSK9/J16 internalization. Furthermore, an anti-APLP2 antibody, 12E3, that specifically blocks PCSK9 binding (S1B Fig; measured at pH 6.0) significantly inhibited PCSK9-488/J16 complex internalization in HepG2 cells (Fig 3A and 3B). These results directly support our siRNA findings, and together indicate that APLP2, but not APP, mediates PCSK9 internalization in HepG2 cells when PCSK9 cannot bind LDLR.

During these studies, we also observed that PCSK9-488/5F6 complex endocytosis was attenuated in *APP* knockdown cells (29.4±3.3% inhibition; Fig 3C and 3D). This effect may be attributed to the transcriptional suppression and corresponding diminished LDLR protein levels in *APP* knockdown cells (S1A Fig) [14]. We hypothesize that in the absence of APLP2 binding, PCSK9 relies on LDLR binding for endocytosis, and thus PCSK9/5F6 complex internalization correlates strongly with LDLR levels. To determine whether LDLR binding is indeed required for APLP2 independent endocytosis, we utilized an LDLR specific antibody, RD-LDLR, which inhibits LDLR interactions with PCSK9 (S1C Fig). Importantly, RD-LDLR blocked internalization of PCSK9-488/5F6 complexes, but not PCSK9-488 alone (Fig 3C and 3D). Together, these data indicate that LDLR and APLP2 are each sufficient to mediate PCSK9 endocytosis, and that PCSK9 internalization in hepatic cells can be blocked by inhibiting both of these interactions.

### LDLR facilitates PCSK9 interactions with APLP2 at neutral pH

We previously reported that we did not detect PCSK9/APLP2 interactions by co-immunoprecipitation (coIP) at neutral pH, a finding that is inconsistent with APLP2 playing a direct role in PCSK9 endocytosis [14]. Importantly, lysates for that study were prepared from cells that were harvested using Accutase, which has proteolytic activity and could affect surface exposed proteins. We hypothesized that PCSK9 interacts with a distinct cell surface population of APLP2 that is sensitive to Accutase treatment. Indeed, PCSK9/J16 coIPs from HepG2 cells that were directly lysed revealed that APLP2 and PCSK9 can interact at neutral pH, and this interaction is sensitive to the proteolytic activity of Accutase (S2A Fig).

**Fig 4:**
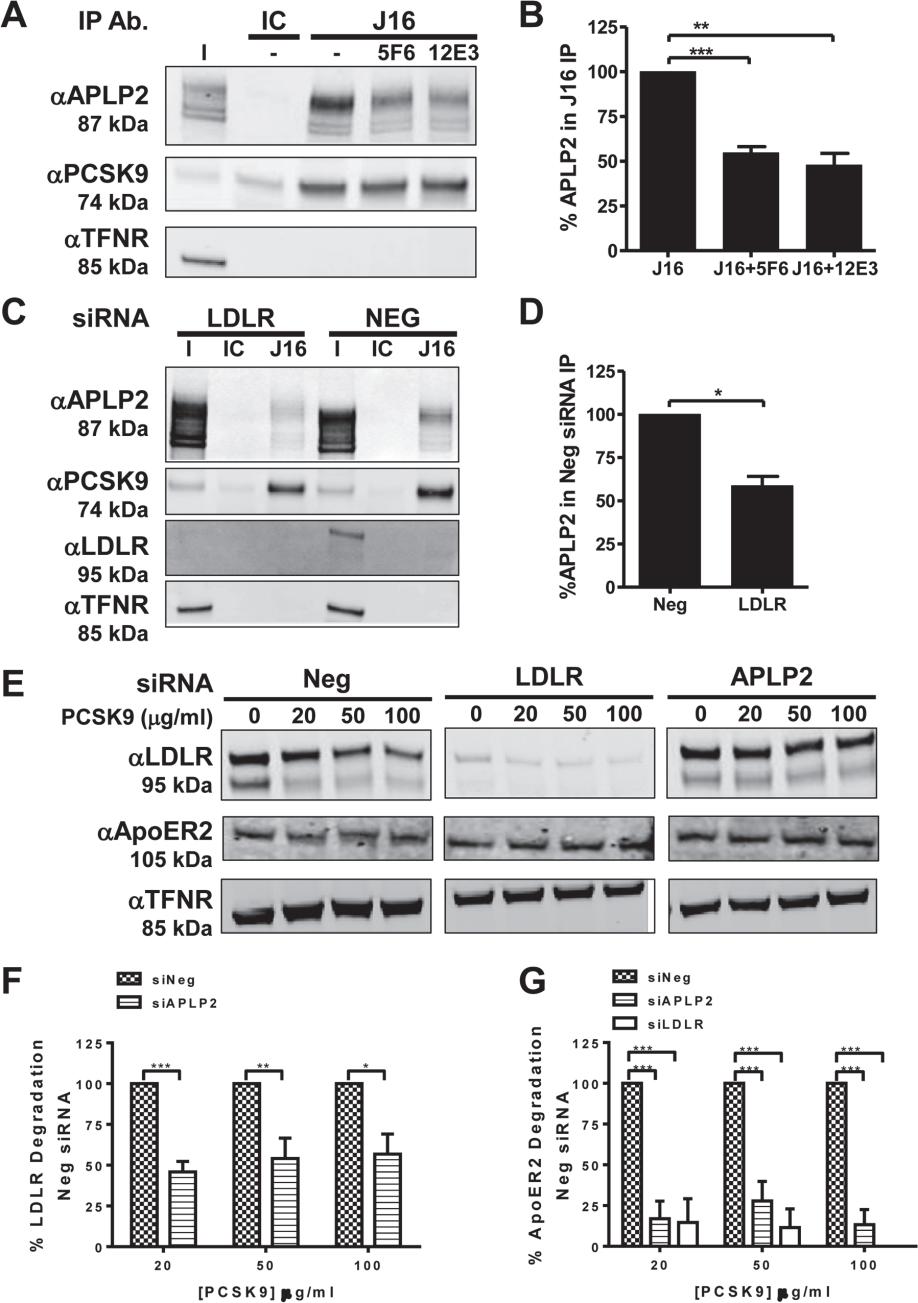
APLP2 and LDLR interactions with PCSK9 and their regulation of PCSK9 function. (A and B) Western blot showing APLP2, PCSK9, or Transferrin receptor (TFNR) levels in input fraction (I), IC or J16 immunoprecipitated samples (IP Ab.) in the absence or presence of 5F6 Fab or 12E3 Fab, as indicated. (B) Quantification of (A); shown as average APLP2 normalized to PCSK9 of 3 independent experiments with SEM. (C and D) J16 coIPs of PCSK9 from Neg or LDLR siRNA treated HepG2 cells with IC control, as indicated. (D) Quantification of (C); shown as average APLP2 normalized to PCSK9 from 3 independent experiments with SEM. (E, F, and G) Western blot of LDLR, APOER2, or TFNR in siRNA treated cells following treatment with PCSK9 at 0, 20, 50, or 100 μg/ml. (F) LDLR levels from (E) quantified as percent LDLR degradation of untreated cells and normalized to Neg siRNA samples. Shown as average with SEM from 4 independent experiments. (G) Same as F, but measuring APOER2 levels.

To determine the specificity of this interaction, PCSK9/J16 coIPs were performed in the presence of 5F6 Fab or 12E3 Fab, which reduced coIPed APLP2 levels by 45.2±3.5% and 52.1±6.5%, respectively (Fig 4A and 4B). However, we were unable to detect a direct interaction between PCSK9 and APLP2 using recombinant proteins at neutral pH, possibly due to the lack of other co-factors or specific modifications on APLP2 that may be required for this interaction to occur.

We also observed that LDLR interacts with APLP2 at neutral pH (S2A Fig). This interaction is direct, since recombinant APLP2-ECD bound LDLR-ECD by ELISA at pH7.4 (S2B Fig). Furthermore, the presence of PCSK9 is not required for LDLR to bind APLP2 by ELISA (S2C Fig), and 5F6 Fab and/or J16 Fab do not affect the complex in HepG2 cell lysates at pH 7.4 (S2D Fig). Thus, contrary to our findings at pH 6.0 [14], PCSK9 does not facilitate APLP2/LDLR interactions at neutral pH.

Given these data and our previous finding that expression of LDLR is required for PCSK9 endocytosis even in the absence of a direct LDLR interaction [14], we hypothesized that LDLR facilitates APLP2-mediated PCSK9 internalization. To address potential mechanisms by which this occurs, we observed APLP2 internalization and PCSK9 interactions in *LDLR* siRNA treated cells. APLP2 is internalized efficiently in *LDLR* knockdown cells, thus, APLP2 does not require LDLR for its own internalization (S3A and S3B Fig). We next looked at PCSK9/APLP2 binding in LDLR depleted cells to determine whether LDLR is involved in this interaction at neutral pH. Indeed, in coIPs of PCSK9/J16 from lysates of *LDLR* knockdown cells, levels of APLP2 normalized to levels of immunoprecipitated PCSK9 were 41.3±5.4% lower than what was observed in negative siRNA control coIPs (Fig 4C and 4D). Notably, no detectable LDLR is seen in J16/PCSK9 coIPs at neutral pH in the negative siRNA control indicating that the APLP2/LDLR interactions may be weak and transient under these conditions and confirming that PCSK9 cannot interact with LDLR when J16 is present. Together, these data indicate that LDLR is involved in PCSK9/APLP2 interactions at neutral pH, a finding that supports a role for LDLR in APLP2-mediated PCSK9 internalization.

To understand if LDLR and APLP2 are generally required for PCSK9 function, we examined whether they influence the ability of PCSK9 to degrade APOER2, a known PCSK9 target. Previously we showed that APLP2 is required for PCSK9-mediated LDLR degradation by directing the complex to lysosomes [14]. To determine whether LDLR and APLP2 expression are required for internalization and lysosomal delivery of APOER2, we measured PCSK9-mediated degradation in *LDLR* or *APLP2* knockdown cells. The siRNA oligos used in this experiment were previously validated for specificity in PCSK9 degradation assays [14]. In these assays, we used saturating concentrations of PCSK9 in an effort to overcome threshold effects from the residual levels of LDLR or APLP2 in the cells. As expected, PCSK9 mediated LDLR degradation was significantly attenuated in *APLP2* knockdown cells relative to control cells (Fig 4E and 4F). In negative control cells, APOER2 was degraded in a manner comparable to what has been previously reported in the literature [8]. Importantly, APOER2 degradation was inhibited in both *LDLR* and *APLP2* knockdown cells over a series of PCSK9 concentrations (Fig 4E and 4G). These results indicate that LDLR and APLP2 are each required for efficient PCSK9-mediated degradation of APOER2, and may therefore be generally required for PCSK9 function.

### LDL can indirectly facilitate PCSK9 internalization

Thus far, our studies have been limited to assays devoid of lipoproteins. Intriguingly, recent studies have shown that 25–40% of plasma PCSK9 is bound to LDL, and that LDL plays a role in PCSK9 clearance [27–29]. One such report showed that physiological concentrations of LDL inhibit PCSK9 internalization and subsequent LDLR degradation. Notably, the authors of this study also found that PCSK9 does not affect LDL/LDLR interactions [29], suggesting the possibility of ternary complex formation between these players.

**Fig 5:**
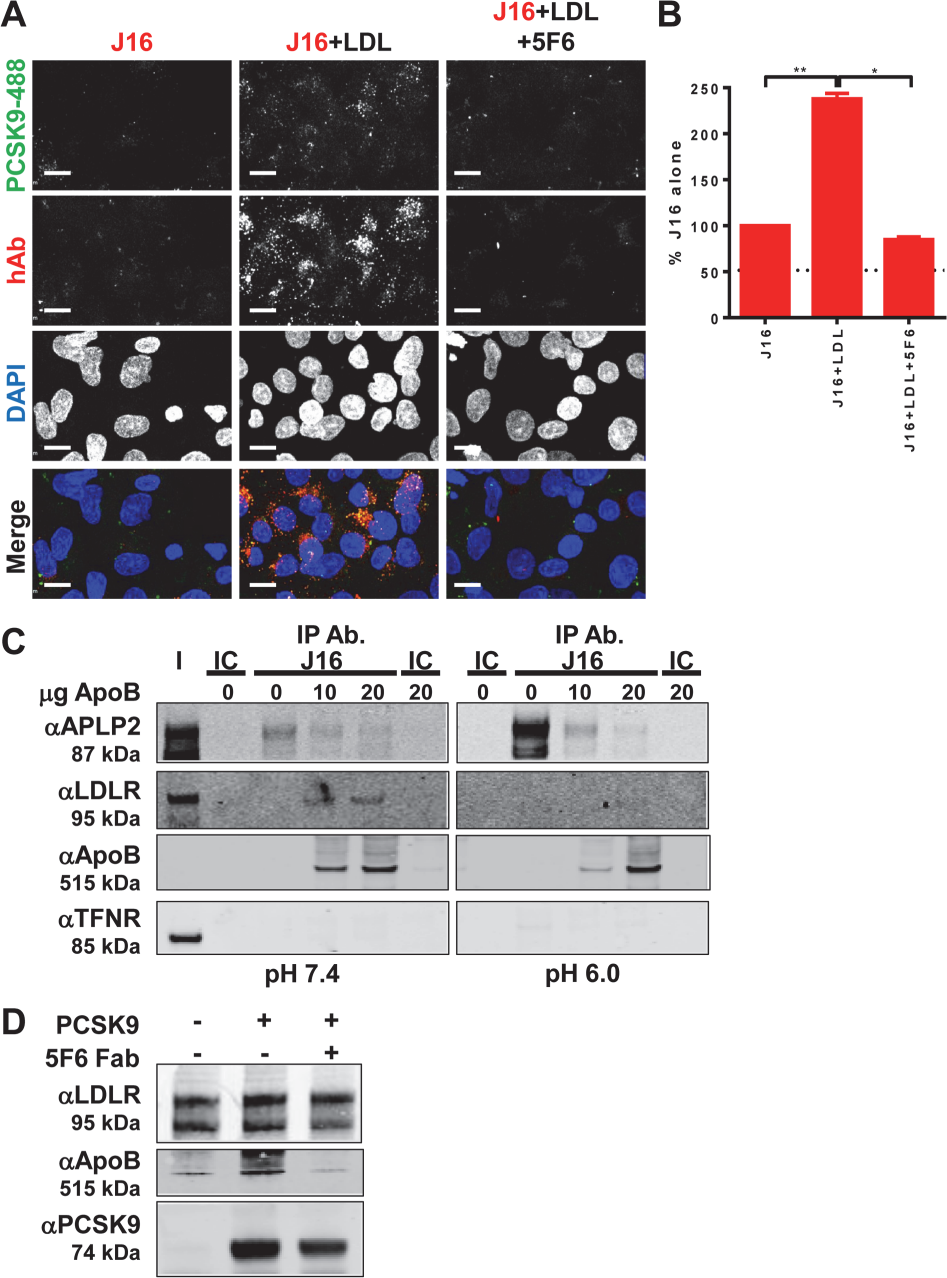
ApoB/LDL effects on PCSK9 internalization and function. (A and B) PCSK9-488 internalization in the presence of J16 (top row), LDL and J16 (middle row), or LDL+5F6+J16 (bottom row) in APLP2 siRNA treated cells. Dotted line indicates background signal, as measured by IC alone. Scale bars, 10 μM. (B) Quantification of (A) shown as average+SEM of J16 fluorescent signal per cell in APLP2 siRNA treated cells from 3 independent experiments. Dotted line indicates average IC background levels. Scale bars, 10 μM. (C) Representative western blot showing APLP2, LDLR, ApoB, Transferrin receptor (TFNR) levels in input fraction (I), IC or J16 immunoprecipitated samples (IP Ab.) under pH 7.4 or pH 6.0 conditions with increasing concentrations of ApoB, as indicated. (D) Western blot showing recombinant ApoB, LDLR-ECD, or PCSK9 in anti-LDLR immunoprecipitated samples at pH 6.0, with or without 5F6 Fab, as indicated. All experiments were performed independently at least 3 times and representative data are shown here.

Based on these reports, we tested whether LDL can indirectly mediate PCSK9 internalization by bridging it to LDLR. To assess this, we eliminated the direct routes by which PCSK9 can be internalized. PCSK9-488 was first bound to J16 to eliminate LDLR interactions, and the PCSK9-488/J16 complexes were subjected to internalization assays in *APLP2* siRNA treated cells. As expected, under these conditions, there was minimal internalized PCSK9-488 (Fig 5A). The presence of 2.5 μg/ml LDL, however, increased PCSK9 internalization by roughly 2.5 fold (Fig 5A and 5B). Surprisingly, addition of 5F6 inhibited LDL-mediated PCSK9 internalization, indicating that 5F6 blocks binding of LDL to PCSK9, and that LDL and APLP2 compete for PCSK9 binding (Fig 5A and 5B). In further support of this, recombinant ApoB, the protein component of LDL that binds LDLR, added to HepG2 cell lysates was co-immunoprecipitated by PCSK9/J16 and also inhibited APLP2 interactions with PCSK9 in a dose-dependent manner. ApoB interacted with LDLR under these conditions, as evidenced by the ApoB dependent presence of LDLR (Fig 5C). Thus, ApoB directly competes with APLP2 for direct interactions with PCSK9 and can simultaneously bind LDLR at neutral pH.

At pH 6.0, ApoB similarly competed with APLP2 in PCSK9/J16 coIPs, despite the increased affinity of PCSK9 for APLP2 at endosomal pH (Fig 5C). Notably, there was no change in the amount of ApoB coIPed, suggesting that its interaction with PCSK9 is not sensitive to changes in pH. ApoB has a well-described pH dependent relationship with LDLR, and as expected, was unable to efficiently bind LDLR under these conditions (Fig 5C). These data together with our previous finding that PCSK9 bridges APLP2 to LDLR at pH 6.0 [14], led us to the hypothesis that PCSK9 may similarly bridge ApoB/LDL to LDLR at endosomal pH. Indeed, recombinant PCSK9 added to recombinant LDLR-ECD correlated with an increase in the presence of ApoB in coIPs performed at pH 6.0. This direct interaction was reversible with 5F6 Fab, indicating that, similar to what was observed at pH 7.4, it is specific and utilizes the APLP2 binding epitope on PCSK9 (Fig 5D). These findings reveal that there are multiple competitive internalization routes for PCSK9, which may in turn contribute to varying downstream trafficking events.

### PCSK9 internalization *in vivo*

**Fig 6:**
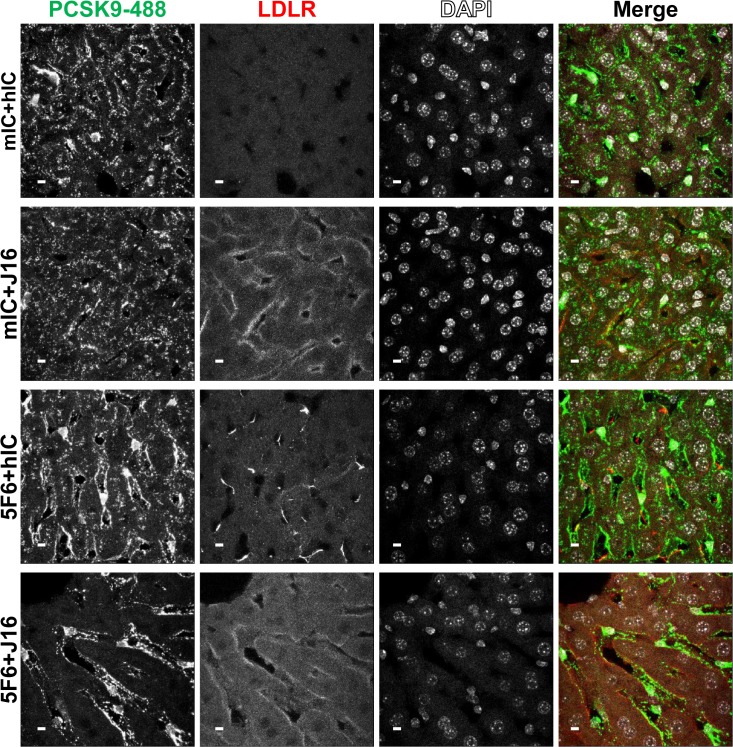
PCSK9-488 internalization and LDLR levels *in vivo*. PCSK9-488 (green) internalization in mouse liver in the presence of mIC and hIC (top row), mIC and J16 (middle top row), hIC and 5F6 (middle bottom row), or 5F6 and J16 (bottom row). LDLR (red) and DAPI (blue) staining shown, as indicated. Scale bars, 10 μM.

To address these internalization pathways under more physiological conditions, we next established an *in vivo* model for PCSK9 internalization. Consistent with our *in vitro* data, J16 complexed with recombinant human PCSK9 injected intravenously in wild type *C57BL/6* mice was visibly internalized in hepatocytes 1 hr after injection, while no visible internalized human antibody was observed in J16 alone, hIC alone, or hIC combined with PCSK9 under these conditions (S3C Fig). To compare *in vitro* internalization requirements *in vivo*, *Pcsk9* ^*-/-*^ mice were injected with PCSK9-488 pre-mixed with hIC+mIC, J16+mIC, hIC+5F6 or J16+5F6. PCSK9-488 was readily taken into hepatocytes when coinjected with IC, 5F6, or J16 alone. However, PCSK9-488 was not internalized and remained in the sinusoids between hepatocytes when both endocytic epitopes were blocked by J16 and 5F6 (Fig 6).

**Fig 7:**
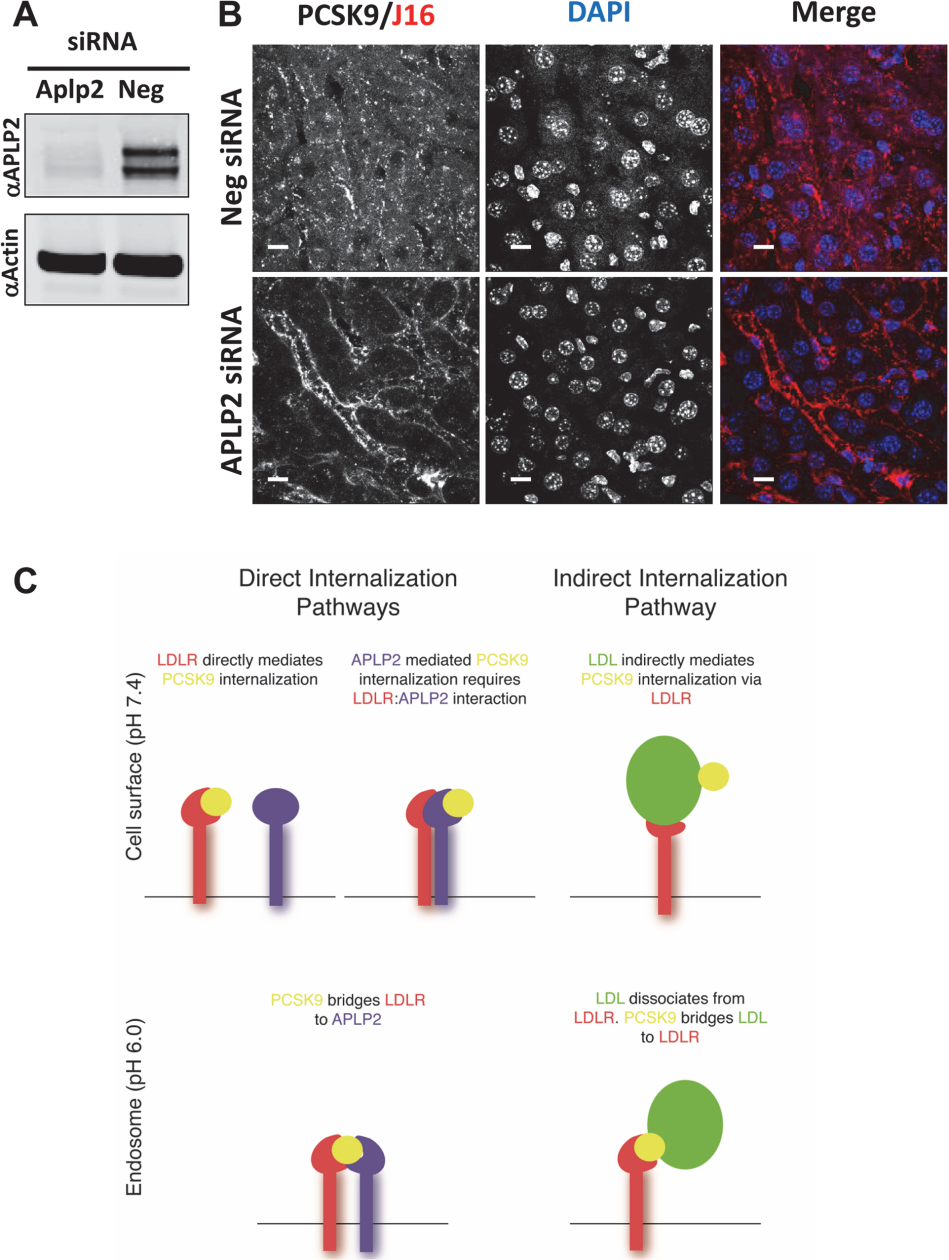
PCSK9 follows both direct and indirect internalization routes. (A) Western blot of liver lysates taken from negative siRNA or APLP2 siRNA treated mice showing relative APLP2 levels as compared to Actin loading control. (B) Internalization of J16 bound PCSK9 in liver in negative or APLP2 siRNA treated mice. Scale bars, 10 μM. (C) Schematic depicting interactions of the proposed direct and indirect PCSK9 internalization routes. At the cell surface, PCSK9 can bind directly to LDLR or APLP2; PCSK9 binding to APLP2 requires LDLR/APLP2 interactions. For both direct routes, following endocytosis, PCSK9 bridges LDLR to APLP2, and APLP2 mediates lysosomal delivery of the complex. Indirect PCSK9 internalization is mediated via LDL. PCSK9 binds LDL, and LDL binds LDLR at the cell surface. Following endocytosis, PCSK9 can potentially bridge dissociated LDL to LDLR.

We next utilized siRNA oligos to knock down expression of APLP2 in Pcsk9^-/-^ mice to specifically determine whether PCSK9/J16 internalization is APLP2 dependent *in vivo*. Importantly, liver APLP2 levels were significantly diminished in APLP2 siRNA treated mice relative to negative control siRNA treated mice (Fig 7A), and this loss of APLP2 correlated with a strong attenuation of PCSK9/J16 complex internalization (Fig 7B). Interestingly, some PCSK9/J16 internalization was observed, which could be due either to lipoprotein mediated internalization or incomplete knockdown of APLP2. Overall, these data support our hypothesis that APLP2 is required for PCSK9 internalization when LDLR binding is unavailable, both *in vitro* and *in vivo*.

PCSK9 injected intravenously into mice has been shown to degrade hepatic LDLR [30, 31]. Thus, as expected, liver LDLR was almost completely degraded in PCSK9-488+mIC+hIC treated mice, while J16 or J16+5F6 inhibited PCSK9-mediated LDLR degradation completely. Interestingly, 5F6 alone only partially attenuated LDLR degradation (Fig 6). Incomplete protection may be due to insufficient affinity of 5F6 for PCSK9 or PCSK9 merging with a previously described intracellular route [32] and mediating degradation of LDLR independently of the endocytic pathways. Importantly, the c-terminal domain of PCSK9, which is required for APLP2 interactions and LDLR degradation via the endocytic pathway, is not required for the intracellular route [10]. APLP2 is therefore likely not involved in the intracellular route, in which case 5F6 would not be effective in blocking PCSK9 from degrading LDLR that intersects with this route.

## Discussion

Previously, we showed that PCSK9 is efficiently internalized in hepatic cells regardless of LDLR binding [14]. We also found in that study that PCSK9 endocytosis is dependent on expression of LDLR, even in the absence of a direct interaction. Other groups have shown a relationship between PCSK9 and LDL in which a large proportion of circulating PCSK9 is associated with LDL, and that LDL modulates PCSK9 internalization and clearance [27–29]. Moreover, Kosenko et al. determined that PCSK9 can simultaneously bind to LDL and LDLR. It is therefore possible that LDL indirectly mediates PCSK9 internalization via the LDL/LDLR endocytic route. Based on these studies, we inferred that there are multiple mediators and regulatory mechanisms of PCSK9 endocytosis, which we sought to elucidate in this current study.

To dissect potential roles of the PCSK9 binding partners, LDLR, APP, and APLP2, in its endocytosis, we subjected our unique toolset of blocking antibodies to a previously validated internalization assay. These experiments were done in the absence of lipoproteins to avoid any confounding effects of PCSK9 interactions with LDL. Our data indicated that APLP2, a protein that mediates PCSK9 lysosomal delivery, is a direct and sufficient mediator of PCSK9 internalization in the absence of LDLR binding. In a complementary manner, a direct LDLR interaction is required for PCSK9 internalization when APLP2 is unavailable. LDLR and APLP2 utilize distinct binding epitopes on PCSK9 that can be simultaneously blocked to completely inhibit PCSK9 internalization. Thus, APLP2 and LDLR binding are each sufficient, while at least one of these partners is necessary, for mediating lipoprotein-free PCSK9 endocytosis (Fig 7C).

During these studies, we determined that LDLR interacts directly with APLP2, and loss of LDLR expression results in decreased APLP2 interactions with PCSK9. We hypothesize that LDLR induces a conformational change in APLP2 that facilitates PCSK9 binding at the cell surface (Fig 7C), or alternatively, LDLR delivers a third, as of yet unknown binding partner to mediate this interaction. LDLR regulation of APLP2-mediated PCSK9 interactions and their downstream internalization makes LDLR necessary for PCSK9 endocytic events, even without directly binding PCSK9. In further support of LDLR playing a general role in PCSK9 function, knockdown of *LDLR* inhibits PCSK9-mediated APOER2 degradation. We previously showed that APLP2 mediates PCSK9/LDLR lysosomal delivery and degradation (Fig 7C), and here we show that it is similarly required for APOER2 degradation. Thus, LDLR and APLP2 are both critical for general PCSK9 function, and likely work together to mediate its various trafficking events.

Another possible mediator of PCSK9 function is LDL, which has been shown to interact directly with PCSK9 and influence its uptake [27–29]. We tested whether LDL bound PCSK9 (PCSK9/LDL), which is a predominant and active species of PCSK9 in circulation, can be internalized via LDL binding to LDLR. Indeed, at low concentrations where free LDL does not compete with PCSK9/LDL for LDLR binding [29], LDL facilitated PCSK9 internalization, presumably by bridging it to LDLR (Fig 7C). Surprisingly, 5F6 effectively blocked this internalization route, indicating that LDL and APLP2 share an epitope on the c-terminal domain of PCSK9. Indeed, ApoB competes with APLP2 for PCSK9 binding, both at pH 7.4 and pH 6.0.

Intriguingly, unreleased LDL could provide an alternative route by which PCSK9 facilitates LDLR lysosomal delivery. ApoB has a well-understood, pH dependent relationship with LDLR that allows LDL to be released from LDLR upon exposure to endosomal pH. The released LDL particle continues on to lysosomes, while LDLR is recycled back to the cell surface to repeat the process. This relationship is critical for LDLR trafficking, as loss of this release mechanism forces LDL bound LDLR to lysosomes for degradation [33]. Our data show that PCSK9 can physically bridge APOB to LDLR at endosomal pH, indicating that PCSK9 could form a complex with LDL and LDLR in endosomes (Fig 7C). Such PCSK9-mediated LDL reassociation with LDLR in lysosomes could provide an APLP2 independent means by which PCSK9 degrades LDLR.

While interesting, an LDL mediated internalization mechanism may be highly inefficient as PCSK9/LDL complex uptake likely depends on the relative molar ratio of PCSK9 to LDL. Under high, physiological LDL:PCSK9 ratios, PCSK9 bound LDL particles would be in competition with free LDL particles, which in turn would limit the internalization efficiency of PCSK9. Consistent with this, Kosenko et al. show a significant inhibition of PCSK9 uptake in the presence of increasing, physiological levels of LDL. Thus, we hypothesize that PCSK9 predominantly enters cells through LDLR and/or APLP2 direct binding.

The regulatory role of LDLR in its own degradation or the degradation of other targets is also likely proportional to its expression levels. At low LDLR levels and/or high LDL levels, we hypothesize that PCSK9 activity is attenuated because (1) it has less opportunity to bind LDLR, and (2) LDLR is not able to readily mediate APLP2 interactions with PCSK9 at neutral pH. This in turn provides less opportunity for PCSK9 mediate degradation of LDLR and its other targets. At high levels of LDLR and or/low serum LDL levels, the opposite would be true. Thus, the stimulus that drives PCKS9 mediated degradation may simply be the relative ratios LDLR, PCSK9, and LDL protein levels, while the interactions between all of these players determine its trafficking routes and subsequent degradation.

Here, we show that PCSK9 can be internalized in hepatic cells directly by APLP2 or LDLR, or indirectly through interactions with ApoB/LDL. Interestingly, all three of the PCSK9 internalization mechanisms described in this study can be blocked by a combination of 5F6 and J16, as shown both *in vitro* and *in vivo*. Altogether, this study defines distinct, conserved routes for internalization of PCSK9, and indicates that PCSK9 may carry out its functions through a multitude of mechanisms.

## Experimental Procedures

### Protein Purification

Recombinant human PCSK9, isotype control antibodies (mIC and hIC), and J16 were purified exactly as in [22]. 5F6 and APLP2-ECD were purified exactly as in [14]. 12E3 was identified in an anti-APLP2-ECD antibody screen from hybridoma cultures according to methods described in [22] and purified using monoclonal antibody select protein A beads (GE Healthcare, Pittsburgh, PA). LDLR-ECD-6xHis was cloned into pAPLP2ECD [14] using the Nhe1/Age1 sites, and purified using standard techniques. PCSK9 was labeled with Alexa Fluor 488, according to manufacturer's instructions (Life Technologies, Carlsbad, CA) with an average of 2 dye molecules per PCSK9 molecule (PCSK9-488).

### Cell Culture and siRNA knockdown

HepG2 cells were cultured in DMEM supplemented with 10% FBS, L-glutamine, and pen-strep. For siRNA knockdown, HepG2 cells were transfected using RNAiMax lipofectamine reagent and Ambion silencer select siRNA oligos (Life Technologies), according to manufacturer’s reverse transcription protocol.

### Internalization assays and immunofluorescence

Assays were conducted as in [14]. Media was exchanged for DMEM with 10% lipoprotein deficient serum (LPDS) at least 16 hours prior to assays. 7.5 μg/ml PCSK9-488 was premixed with 25 μg/ml of antibodies or Fab fragments and added to HepG2 cells or primary mouse hepatocytes plated on uncoated or collagen coated glass coverslips, respectively. Fab fragments were produced by digesting IgG molecules with Pierce mouse IgG1 Fab Digestion Kit (Thermo-Fisher Scientific, Rockford, IL). LDL (Life Technologies) was added at 2.5 μg/ml in indicated assays. A control for internalization was performed by binding PCSK9-488 to cells at 4°. After a 3 hour incubation at 37 degrees, cells were fixed with 4% formaldehyde, permeabilized with 0.2% Triton X-100, and blocked. 2 μg/ml goat anti-mouse IgG1 647 secondary antibody, goat anti-Human 546, donkey anti-mouse 647, or donkey anti-goat 546 secondary antibodies (Life Technologies) were used to detect internalized antibodies. siRNA internalization assays were performed identically 72 hours following transfection.

For APLP2 internalization, HepG2 cells were incubated with anti-APLP2 monoclonal antibody (R&D systems, Minneapolis, MN) for 30 minutes at 4 degrees. Cells were washed twice with cold media, shifted to warm media, and incubated at 37 degrees for 15 minutes. Cells were then fixed, permeabilized, blocked, and stained for internalized mouse antibody (goat anti-mouse IgG2b 488; Life Technologies) before being subjected to confocal microscopy.

### Mouse studies

All animal maintenance and handling in this study were conducted and approved in accordance with approved protocols by the Institutional Animal Care and Use Committee (IACUC) at Pfizer Inc in an AAALAC accredited facility.

6–10 week old PCSK9^-/-^ or wild type *C57BL/6* mice were purchased from Jackson Laboratories. Mice were housed in micro-insulator units with free access to standard chow and water and were acclimated to the facility for at least 10 days prior to start of experiments.

8–12 week old male *C57BL/6* mice weighing approximately 20 grams were used to isolate primary hepatocytes. After mice were anesthetized with xylene (~15 mg/kg) and ketamine (~50 mg/kg), livers were perfused through the hepatic portal vein with Hepatocyte Perfusion Buffer (Life Technologies) until blood cleared through the inferior vena cava. Livers were then perfused with 25mL of collagenase solution (1 mg/mL collagenase (Sigma) in Hepatocyte Wash Buffer (Life Technologies). Livers were extracted and filtered through a 70μm filter. Cells were washed twice and resuspended in 20% Histodenz (Sigma) to collect hepatocytes. After centrifugation, hepatocytes were resuspended in Hepatocyte Wash Buffer with 5% FBS and 1% P/S. ~3x10^5 cells were seeded onto collagen-coated coverslips BD Bioscience) in 6-well plates and left for 1–2 hours at 37°C in 5% CO_2_. Media was replaced with SFM (Life Technologies) with 1% P/S, 1% L-glutamine. Cells were used within 24 hours.

For *in vivo* siRNA knockdown, complexes of APLP2 or negative control *in vivo* siRNA oligos (Ambion; Life Technologies) were prepared using Invivofectamine exactly according to manufacturer’s instructions (Life Technologies) and injected by tail vein into age and gender matched Pcsk9 ^-/-^ mice 24 hours prior to *in vivo* internalization assays.

For *in vivo* internalization assays, IC, J16, or 5F6 (15 mg/kg) alone or premixed with PCSK9 or PCSK9-488 (3 mg/kg) were injected by tail vein into age and gender matched Pcsk9 ^-/-^ or wild type mice. After 1 hour, mice were anesthetized with xylene (15 mg/kg) and ketamine (50 mg/kg) and livers were perfused through the hepatic portal vein with PBS until the blood cleared, harvested, and fixed in 4% PFA. After incubating in 40% sucrose overnight, livers were embedded in OCT and flash frozen. 12 μm sections were prepared by cryostat sectioning (CM1850, Leica).

### Immunofluorescence of liver sections

Liver sections were incubated in blocking buffer (0.3% TX-100, 300mM Glycine, 2 mg/ml BSA, 10% goat or donkey serum in PBS-T) for at least 1 hour, followed by incubation overnight at 4 degrees with 3 μg/ml anti-Alexa-488 antibody (Life Technologies) or LDLR antibody (R&D Systems) diluted in incubation buffer (0.3% Triton x-100, 2% BSA, 1% serum, PBS-T). For visualizing internalized antibodies, anti-human secondary antibody was applied for 2 hours at room temperature. All secondary antibodies were from Life Technologies and diluted 1:1000. Sections were mounted using Prolong Gold with DAPI (Life Technologies) and imaged by confocal microscopy.

### Confocal Microscopy

Microscopy images from z stacks with 0.5 μm increments were collected using a 63x, 1.4NA objective lens at room temperature on a Leica SP3 laser scanning confocal microscope (Leica, Buffalo Grove, IL). All images shown are projections of optical sections. Data analysis was performed using Leica LAS AF software. Internalization was quantified as intensity of fluorescence signal per cell, from at least 45 cells for each of 3 experiments. Average intensity was normalized against negative control or IC, as indicated in figure legends.

### CoImmunoprecipitation

CoIPs were performed similarly as in [14]. Briefly, cells were removed from plates by Accutase treatment and then lysed in coIP buffer or alternatively cells were directly lysed on the plate with coIP buffer (20mM Hepes, 7.4 or 6.0, 20mM CaCl2, 150mM NaCl, 0.1% Triton x-100). Cells were passed through a 27g needle twice and pelleted for 15 minutes at 16,100xg. Lysates were precleared with mAb select protein A beads (GE Healthcare), and then incubated with 10 μg/ml anti-LDLR, anti-APLP2, J16, or IC antibodies and mAb select beads overnight. 10 μg/ml of 12E3 or 5F6 Fab fragments were added to IPs where indicated. Recombinant ApoB (Sigma) was added at indicated concentrations to lysates prior to preclearing step. Beads were washed 3x with coIP buffer and complexes were eluted with sample buffer before running on a 4–12% Bis-Tris gel and transferred to nitrocellulose membranes for western blot analysis. Recombinant coIPs were performed using pH 6.0 coIP buffer with 5 μg/ml ApoB, 5 μg/ml LDLR-ECD, and 5 μg/ml PCSK9. After 1 hour incubation at room temperature, Protein A beads saturated with anti-LDLR were added for 1 hour before wash and elution steps. All coIPs were performed at least 3 independent times.

### PCSK9 sensitivity assay

72 hours after siRNA transfection, 0, 20, 50, or 100 μg/ml PCSK9 was added to HepG2 cells that had been incubated overnight in 10% LPDS media. After 4 hours, lysates were harvested and run onto a 4–12% Bis-Tris gel, and transferred to nitrocellulose membranes for western blot analysis.

### Western blot analysis

Nitrocellulose membranes were blocked with Odyssey blocking buffer (Licor Biotechnologies), incubated with primary antibodies (LDLR (goat anti-Human; R&D Systems), TFNR (mouse anti-Human; Life Technologies), PCSK9 (sheep anti-Human; R&D Systems), APLP2 (goat anti-Human; R&D Systems), APP (goat anti-mouse; Life Technologies), APOER2 (Abcam)) for at least 1 hour, washed, and incubated with secondary antibodies (donkey anti-mouse 680, donkey anti-goat 800, goat anti-rabbit 680, goat anti-mouse 800 (Licor), or donkey anti-sheep 680 (Life Technologies)) before imaging on a Licor Odyssey imaging system. Integrated intensity signals were measured using Odyssey software and normalized against loading controls (as indicated in figure legends).

### ELISA

ELISA of PCSK9 binding to APLP2-ECD or LDLR-ECD was performed exactly as in [14], but using 12E3, J16, or RD-LDLR (R&D Systems) at concentrations indicated in the figure. LDLR/APLP2 ELISA was performed by coating MaxiSorp plates (Thermo-Fisher) with 5 μg/ml LDLR, blocking, and binding APLP2-ECD at the indicated concentrations. PCSK9 was added at indicated concentrations with 5 μg/ml APLP2-ECD. APLP2-ECD was detected using mouse anti-human APLP2 and goat anti-Mouse HRP antibody (R&D Systems), followed by TMB (Thermo-Fisher) according to manufacturer’s instructions.

### Statistical Analyses

Paired, two-tailed Student's t-test of at least 3 experiments was performed to assess statistical significance in all experiments.

## Supporting Information

S1 Fig siRNA knockdown and effect of 12E3 on APLP2/PCSK9 interactions. (A) Representative western blots showing TFNR, APLP2, and APP levels in negative control and siRNA treated cell lysates, as indicated. LDLR western blot shown for APP siRNA. (B) ELISA showing binding of 1 μg/ml biotinylated PCSK9 (Bio-PCSK9) to APLP2 ECD coated plates (coated at 5 μg/ml), with increasing concentrations of the anti-APLP2 antibody 12E3. Shown as average of triplicate samples with SD. (C) ELISA of 1 μg/ml bio-PCSK9 to LDLR ECD coated plates (coated at 5 μg/ml), with increasing RD-LDLR, as indicated. Shown as average of triplicate samples with SD. J16 effect shown as dotted line.
(TIF)

S2 Fig Characterization of LDLR/APLP2 associations. (A) Western blot showing APLP2, PCSK9, or TFNR levels in input fraction (I) or J16, IC, or LDLR IPs following Accutase treatment or direct lysis, as indicated. (B) ELISA showing APLP2-ECD at varying concentrations binding to LDLR-ECD coated plates. Shown as average of triplicate samples with SD. (C) ELISA of APLP2-ECD binding to LDLR-ECD coated plates, with increasing concentrations of PCSK9. Shown as average of triplicate samples with SD. (D) Western blots of APLP2, LDLR, or TFNR in coIPs. I = Input, N = negative control antibody. IP Ab. represents the antibody used for immunoprecipitation. 5F6 or J16 Fab were added as indicated.
(TIF)

S3 Fig APLP2 internalization in LDLR knockdown cells and PCSK9 mediated J16 internalization in mouse liver. (A) APLP2 (green) internalization in negative control (left), LDLR (middle), or APLP2 (right) siRNA treated DAPI (blue) stained HepG2 cells. Scale Bars, 10 μM. (B) Quantification of (A), calculated average fluorescence intensity, normalized against negative control cells. Shown as Average with SEM from 3 independent experiments. (C) Internalization of J16, IC, or J16/PCSK9 in mouse liver. Human antibodies (green), DAPI (blue); scale bars 10 μM.
(TIF)
