# Supplementary figures and images for: Common Proprotein Convertase Subtilisin/Kexin Type 9 (PCSK9) Epitopes Mediate Multiple Routes for Internalization and Function
Source: PLoS One. 2015 Apr 23;10(4):e0125127. doi: 10.1371/journal.pone.0125127 (PMC4408062; doi:10.1371/journal.pone.0125127)

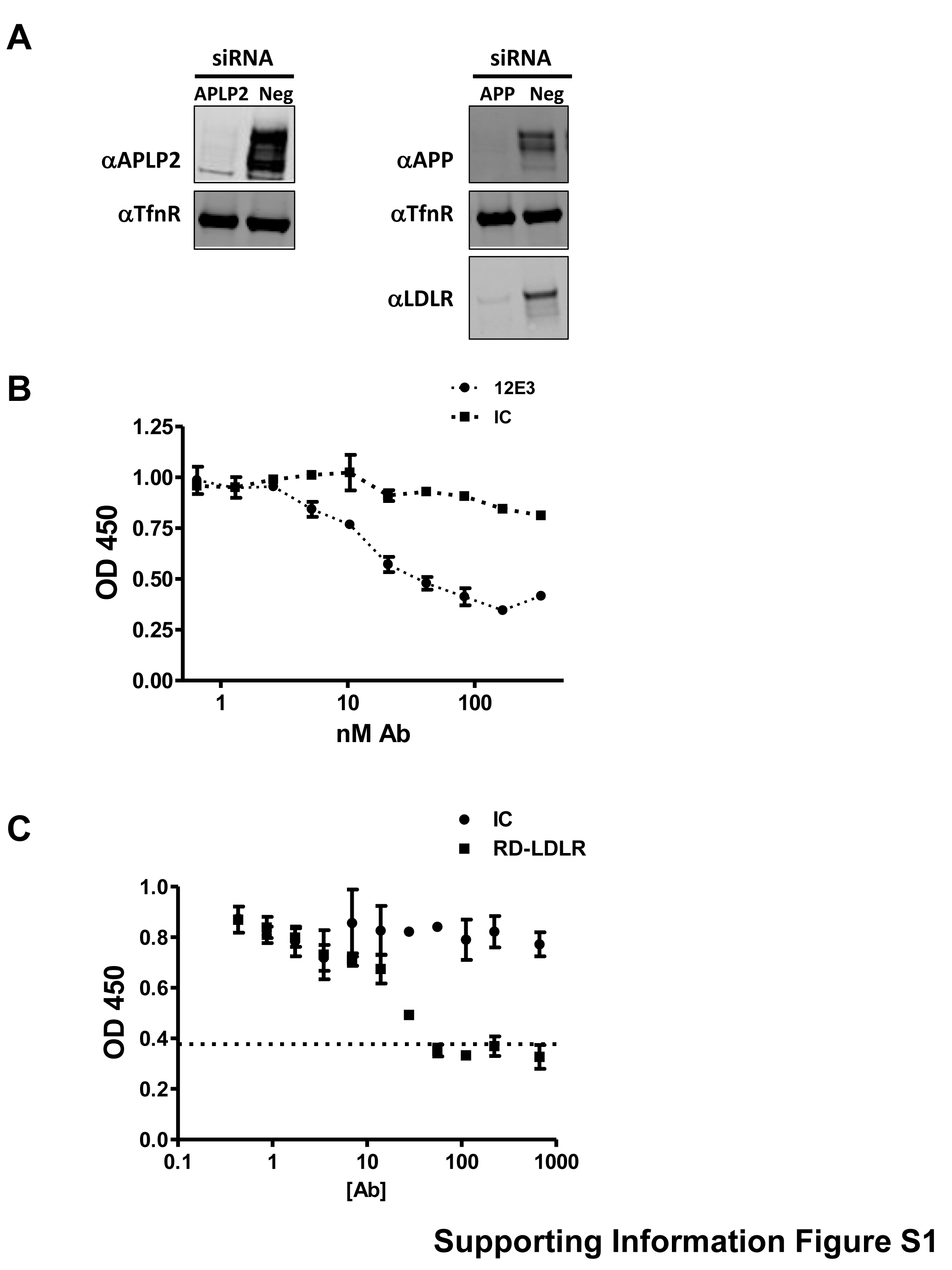

Supplement: S1 Fig — (A) Representative western blots showing TFNR, APLP2, and APP levels in negative control and siRNA treated cell lysates, as indicated. LDLR western blot shown for APP siRNA. (B) ELISA showing binding of 1 μg/ml biotinylated PCSK9 (Bio-PCSK9) to APLP2 ECD coated plates (coated at 5 μg/ml), with increasing concentrations of the anti-APLP2 antibody 12E3. Shown as average of triplicate samples with SD. (C) ELISA of 1 μg/ml bio-PCSK9 to LDLR ECD coated plates (coated at 5 μg/ml), with increasing RD-LDLR, as indicated. Shown as average of triplicate samples with SD. J16 effect shown as dotted line. (TIF) [file pone.0125127.s001.tif]

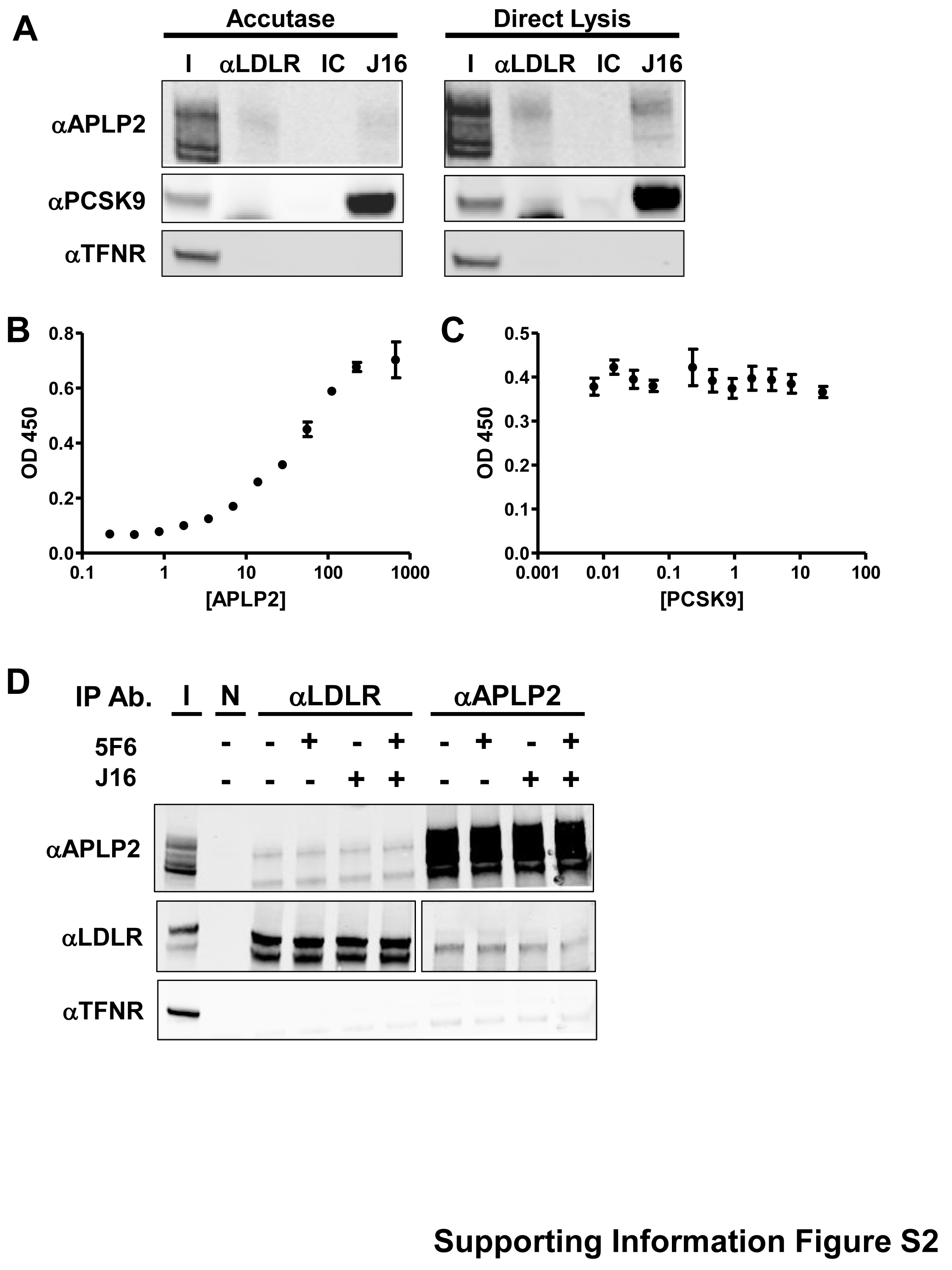

Supplement: S2 Fig — (A) Western blot showing APLP2, PCSK9, or TFNR levels in input fraction (I) or J16, IC, or LDLR IPs following Accutase treatment or direct lysis, as indicated. (B) ELISA showing APLP2-ECD at varying concentrations binding to LDLR-ECD coated plates. Shown as average of triplicate samples with SD. (C) ELISA of APLP2-ECD binding to LDLR-ECD coated plates, with increasing concentrations of PCSK9. Shown as average of triplicate samples with SD. (D) Western blots of APLP2, LDLR, or TFNR in coIPs. I = Input, N = negative control antibody. IP Ab. represents the antibody used for immunoprecipitation. 5F6 or J16 Fab were added as indicated. (TIF) [file pone.0125127.s002.tif]

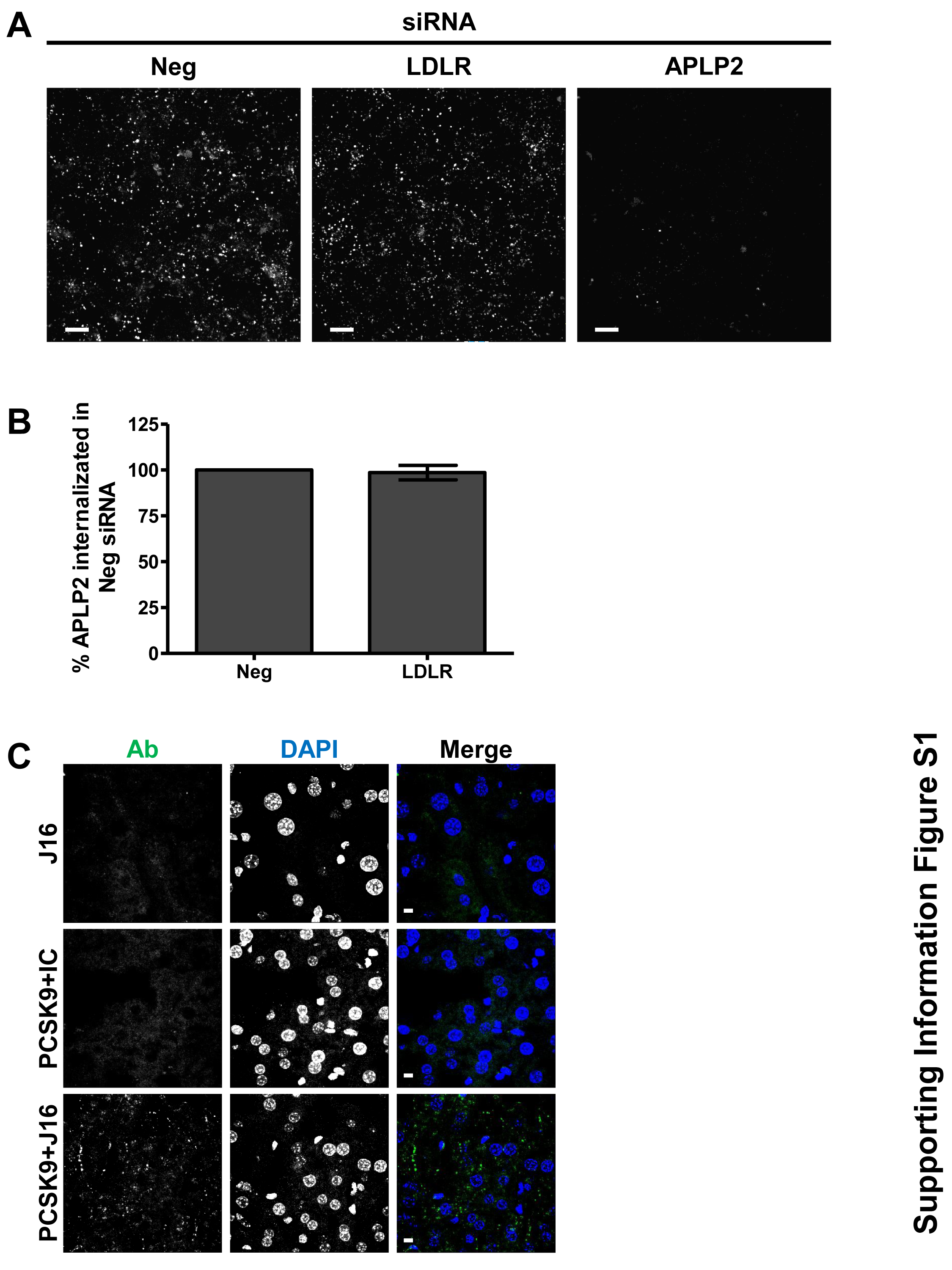

Supplement: S3 Fig — (A) APLP2 (green) internalization in negative control (left), LDLR (middle), or APLP2 (right) siRNA treated DAPI (blue) stained HepG2 cells. Scale Bars, 10 μM. (B) Quantification of (A), calculated average fluorescence intensity, normalized against negative control cells. Shown as Average with SEM from 3 independent experiments. (C) Internalization of J16, IC, or J16/PCSK9 in mouse liver. Human antibodies (green), DAPI (blue); scale bars 10 μM. (TIF) [file pone.0125127.s003.tif]
